# High-Payload Buccal Delivery System of Amorphous Curcumin–Chitosan Nanoparticle Complex in Hydroxypropyl Methylcellulose and Starch Films

**DOI:** 10.3390/ijms22179399

**Published:** 2021-08-30

**Authors:** Li Ming Lim, Kunn Hadinoto

**Affiliations:** School of Chemical and Biomedical Engineering, Nanyang Technological University, Singapore 637459, Singapore; particletechnology.ntu@gmail.com

**Keywords:** curcumin, nanoparticle complex, buccal drug delivery, polysaccharides

## Abstract

Oral delivery of curcumin (CUR) has limited effectiveness due to CUR’s poor systemic bioavailability caused by its first-pass metabolism and low solubility. Buccal delivery of CUR nanoparticles can address the poor bioavailability issue by virtue of avoidance of first-pass metabolism and solubility enhancement afforded by CUR nanoparticles. Buccal film delivery of drug nanoparticles, nevertheless, has been limited to low drug payload. Herein, we evaluated the feasibilities of three mucoadhesive polysaccharides, i.e., hydroxypropyl methylcellulose (HPMC), starch, and hydroxypropyl starch as buccal films of amorphous CUR–chitosan nanoplex at high CUR payload. Both HPMC and starch films could accommodate high CUR payload without adverse effects on the films’ characteristics. Starch films exhibited far superior CUR release profiles at high CUR payload as the faster disintegration time of starch films lowered the precipitation propensity of the highly supersaturated CUR concentration generated by the nanoplex. Compared to unmodified starch, hydroxypropyl starch films exhibited superior CUR release, with sustained release of nearly 100% of the CUR payload in 4 h. Hydroxypropyl starch films also exhibited good payload uniformity, minimal weight/thickness variations, high folding endurance, and good long-term storage stability. The present results established hydroxypropyl starch as the suitable mucoadhesive polysaccharide for high-payload buccal film applications.

## 1. Introduction

The vast therapeutic activities of curcumin (CUR)—a polyphenol extracted from turmeric plants—have been well-established, where antioxidant, antimicrobial, anti-inflammatory, antidiabetic, and anticancer activities of CUR have been successfully demonstrated in vivo [1,2,3]. Numerous human clinical trials on the use of CUR in the management of several chronic diseases (e.g., cardiovascular, metabolic, neurological, cancers) have also shown promising results [4,5]. Not unlike other pharmaceuticals, the oral route represents the most commonly used delivery route of CUR owing to the convenience and cost-effectiveness of oral dosage forms (e.g., tablets, capsules, liquid suspension) for the patients [6]. The oral route, however, produces low CUR systemic bioavailability due to CUR’s low solubility in the gastrointestinal fluid, and due to CUR degradation by first pass metabolism [7,8]. Consequently, high CUR dosages near its toxicity limit are often needed in clinical trials to achieve the intended therapeutic outcomes [9]. 

Amorphization represents one of the most effective solubility enhancement strategy of poorly soluble drugs by virtue of the ability of amorphous drugs to produce a highly supersaturated concentration of the drug upon dissolution [10,11]. The supersaturation generation is attributed to the low energy barrier for dissolution of amorphous drugs as a result of their metastable liquid-like form. The supersaturation generation results in a high kinetic drug solubility, which is multifold higher than the thermodynamic solubility exhibited by the stable crystalline form [12]. Various amorphous CUR formulations exhibiting enhanced CUR solubility have been developed in the form of amorphous solid dispersion [13,14,15], co-amorphous system [16,17,18], and amorphous nanoparticles [19,20,21]. 

Among the amorphous drug formulations, amorphous CUR–polyelectrolyte nanoparticle complex (or nanoplex in short) presented in Lim et al. [19] stands out because of its significantly simpler preparation method. The amorphous CUR nanoplex is prepared by bulk mixing of aqueous CUR solution with chitosan (CHI) solution acting as the oppositely charged polyelectrolyte (PE). Soluble CUR–CHI complexes were formed upon mixing by virtue of their electrostatic interactions. The soluble complexes subsequently form aggregates due to hydrophobic interactions among the bound CUR molecules. The aggregates precipitate upon reaching a critical mass to form the CUR–CHI nanoplex. The restricted mobility of CUR molecules due to the electrostatic binding with CHI prevent them from re-arranging to ordered crystalline structure, resulting in the formation of amorphous CUR [22]. Importantly, the bioavailability enhancement afforded by the CUR–CHI nanoplex has been demonstrated in vivo for wound healing applications [23,24].

While formulating CUR in the form of the CUR–CHI nanoplex can adequately address its low solubility issue in the gastrointestinal fluid, orally administered CUR remains vulnerable to extensive CUR metabolisms in the gut, regardless of the solubility. Thereby, low systemic bioavailability persists [25]. For this reason, parenteral delivery routes of CUR that can be easily administered by the patients themselves, such as transdermal [26,27,28] and buccal [29,30,31] routes, have been explored. Even though the transdermal and buccal delivery routes of CUR can circumvent its gut metabolism issue, the systemic bioavailability remains poor if the native form of CUR is used due to the low solubility of native CUR in the plasma fluid. Therefore, it is imperative that the solubility-enhanced CUR formulation is incorporated into whichever parenteral formulation used. 

For this reason, the present work aimed to develop a buccal delivery system of the amorphous CUR–CHI nanoplex in the form of nanoplex-loaded polymer films. The buccal route for systemic drug delivery relies on transmucosal absorption of drug molecules through mucosal membranes lining the cheeks [32]. In addition to systemic CUR delivery, the buccal film of the CUR–CHI nanoplex can also have applications in local CUR administration in the oral cavity, as CUR is known to be effective in treating various periodontal diseases [33]. Unlike fast-dissolving oral thin film in which the drug payload is released almost instantaneously [34], the buccal film in the present work was prepared using mucoadhesive polysaccharides to facilitate sustained CUR release known to be ideal for its bioavailability enhancement [35]. 

Buccal films containing drug-loaded nanoparticles have been investigated before using a wide range of drugs and materials for both the film and nanoparticle carrier [29,36,37,38,39,40]. The drug payload in these studies, however, were limited to a maximum value of approximately 1 mg of drug per square centimeter of the film. While the reason was not elaborated in these studies, we rationalized that the limit on the maximum drug payload investigated could be attributed to keeping the drug payload at ≤ 1 mg/cm^2^ in order to (1) minimize agglomeration among the nanoparticles and (2) to maintain the physical integrity of the buccal film. The nanoparticle agglomeration could adversely affect the drug content uniformity in the buccal film and the drug release rate [41]. 

A high drug payload is desired in buccal films intended for sustained drug release, as too low payload limits the amount of drug that can be delivered over time. At low drug payload, multiple dosing is needed, which makes the sustained release formulation redundant, and inevitably reduces patients’ compliance to the dosing regimen. Therefore, the objective of the present work was to develop a high-payload mucoadhesive buccal film containing the amorphous CUR–CHI nanoplex (up to ≈ 4 mg/cm^2^), which is capable of producing a sustained CUR release profile. 

In the present work, we investigated the feasibilities of three mucoadhesive polysaccharides as the matrix former for buccal films at high drug payload. They were (1) hydroxypropylmethyl cellulose (HPMC), (2) pre-gelatinized starch, and (3) pre-gelatinized hydroxypropyl starch. The three polysaccharides exhibit well-established mucoadhesiveness, biocompatibility, and film-forming abilities [41]. HPMC represents one of the most widely used mucoadhesive polymers for buccal drug delivery owed to its hydrogel forming ability, which was ideal for producing a sustained drug release profile [42,43]. Starches, on the other hand, have only been recently investigated for buccal drug delivery, despite their well-established film-forming ability [44,45,46]. Starches, nevertheless, have many applications in pharmaceutical formulations, for example, as fillers/binders/disintegrants in oral tablets [47]. 

The abilities of the HPMC and starch films to accommodate the high drug payload were individually examined using the following criteria: (1) high CUR entrapment efficiency, (2) minimal variations in the film’s weight and thickness, (3) good CUR payload uniformity, and (4) high folding endurance signifying physical robustness. In addition, the buccal films ought to be able to produce sustained CUR release profiles at high drug payload. The ideal sustained CUR release profile would be one that follows the zero-order kinetics, where a constant amount of CUR was released from the film as a function of time, resulting in uniform CUR concentrations over time.

The effects of the plasticizer’s type (i.e., glycerol and propylene glycol) and inclusion of adjuvants (i.e., sodium alginate, polyvinyl alcohol) on the HPMC film’s characteristics were investigated. From the results of the above evaluations, the optimal high-payload nanoplex-loaded buccal film formulation was determined. Subsequently, the physical stability of the amorphous CUR–CHI nanoplex in the optimal buccal film formulation was characterized after long-term storage to examine the crystallization tendency of the embedded nanoplex during its shelf life. The amorphous form stability of the nanoplex during storage is crucial for the nanoplex to maintain its solubility enhancement capability.

## 2. Results and Discussion

### 2.1. Physical Characteristics of Amorphous CUR–CHI Nanoplex

**Figure 1:**
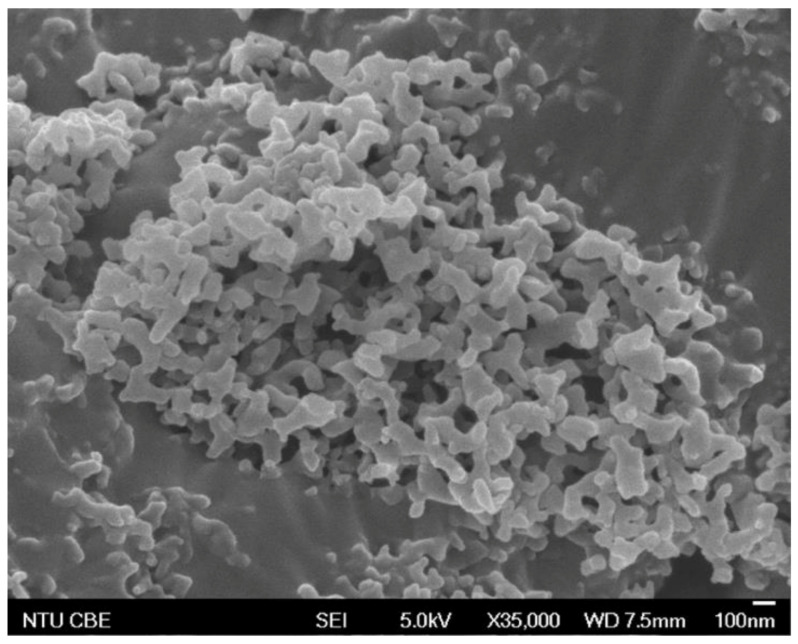
FESEM image of amorphous CUR–CHI nanoplex prior to its incorporation into buccal film.

From DLS, the CUR–CHI nanoplex was found to exhibit size and zeta potential of 244 ± 24 nm and 32 ± 2 mV, respectively. The positive zeta potential indicated the predominant presence of cationic CHI on the nanoplex surface. The CUR content of the nanoplex was 61 ± 2% (*w*/*w*) with CHI making up the remaining mass. The CUR encapsulation efficiency into the nanoplex was 83 ± 3% (*w*/*w*). The FESEM image of the CUR–CHI nanoplex after lyophilization showed the appearance of agglomerates of the nanoplex exhibiting roughly spherical shapes with individual sizes in the range of 100–150 nm (Figure 1). The nanoplex agglomerates shown in the FESEM image readily dissociated into the individual nanoplex upon their reconstitution in deionized water, resulting in similar size and zeta potential (i.e., 258 ± 13 nm and 34 ± 1 mV, respectively).

### 2.2. HPMC-Based Films

#### 2.2.1. Effects of Plasticizer

Physical characteristics of HPMC films prepared using either Gly or PG as the plasticizer are presented in Table 1. The HPMC films were prepared at two theoretical CUR payloads of 1 and 5 mg/cm^2^. As mentioned before, the former represented the typical drug payload used in the previous studies on buccal films of drug nanoparticles, whereas the latter represented the higher drug payload pursued in the present work. 

For HPMC films prepared using Gly as the plasticizer, the effects of increasing the CUR payload on the CUR entrapment efficiency were found to be minimal, with both payloads exhibiting entrapment efficiency of around 81–82% (*w*/*w*). Hence, approximately 20% of the supplied CUR–CHI nanoplex was not successfully embedded into the HPMC matrix during the HPMC film formation. As these free nanoplexes were present on the film’s surface, they were easily removed during the convective drying step. This resulted in experimental CUR payloads of 0.8 ± 0.03 and 4.1 ± 0.3 mg/cm^2^ for the theoretical CUR payloads of 1 and 5 mg/cm^2^, respectively, for HPMC (Gly) films. 

For HPMC films prepared using PG as the plasticizer, the CUR entrapment efficiencies at both payloads were similar in magnitude to the values in the HPMC (Gly) films at around 80% (*w*/*w*). As a result, HPMC (Gly) and HPMC (PG) films exhibited similar experimental CUR payloads. In this regard, as the CUR:CHI ratio in the nanoplex was roughly equal to 60:40 (*w*/*w*), the CHI payloads in the films were equal to approximately two-thirds of the CUR payloads. Therefore, the CHI payloads in the films were equal to roughly 0.5 and 2.6 mg/cm^2^ at theoretical CUR payloads of 1 and 5 mg/cm^2^, respectively.

The similar CUR entrapment efficiency between the HPMC (Gly) and HPMC (PG) films were not unexpected as Gly and PG are highly similar chemically in terms of the alcohol functional groups (3 vs. 2 OH groups), molecular weight (92 vs. 76 g/mol), and density (1.2 vs. 1.0 g/cm^3^). While both Gly and PG are miscible in water, Gly exhibits a significantly higher viscosity (1.41 vs. 0.04 Pa·s). The multifold difference in their viscosities was nevertheless not found to have any impact on the CUR entrapment efficiency. Noticeably, the minimal effect of increased CUR payload on the CUR entrapment efficiency was observed in both the HPMC (Gly) and HPMC (PG) films. These results signified that both HPMC (Gly) and HPMC (PG) films had the capacity to accommodate the larger amount of the CUR–CHI nanoplex at high CUR payload without any adverse effect on the CUR entrapment efficiency. 

While the CUR entrapment efficiency was not affected by the increased CUR payload in the films, the same was not observed for the films’ weight and thickness. The weight and thickness of the HPMC (Gly) and HPMC (PG) films increased from roughly 13–17 mg/cm^2^ and 110–138 µm, respectively, at the low CUR payload to roughly 30–31 mg/cm^2^ and 231–254 µm, respectively, at the high CUR payload (Table 1). The increased thickness at higher CUR payload suggested that the nanoplex accumulated in the films as vertical layers. Importantly, both HPMC films passed the weight and thickness variation tests. All ten independent samples exhibited weight and thickness that were within ± 10% of the average weight and thickness signifying batch-to-batch consistency of their preparation. 

Before the HPMC films were characterized further in terms of their CUR payload uniformity and folding endurance, the CUR dissolution from the films was characterized first to verify that their CUR payload could be released at the desired rate. Being a hydrogel, HPMC films swelled upon dissolution, resulting in the formation of viscous gel layers acting as physical barriers for the CUR release. The CUR release rate from the film was influenced by diffusion of CUR molecules across the gel layers, as well as the CUR dissolution from the CUR–CHI nanoplex. As the CUR–CHI nanoplex was designed as a supersaturating delivery system of CUR, the nanoplex dissolved rapidly in the swollen HPMC matrix to produce a highly supersaturated CUR concentration in the film. Thus, the CUR release rate from the film was essentially governed by the molecular diffusion across the gel layers. Nevertheless, the supersaturated CUR concentration in the film was thermodynamically unstable, thereby, CUR precipitation in the film might take place in the absence of sufficient crystallization inhibition mechanisms [48]. 

**Figure 2:**
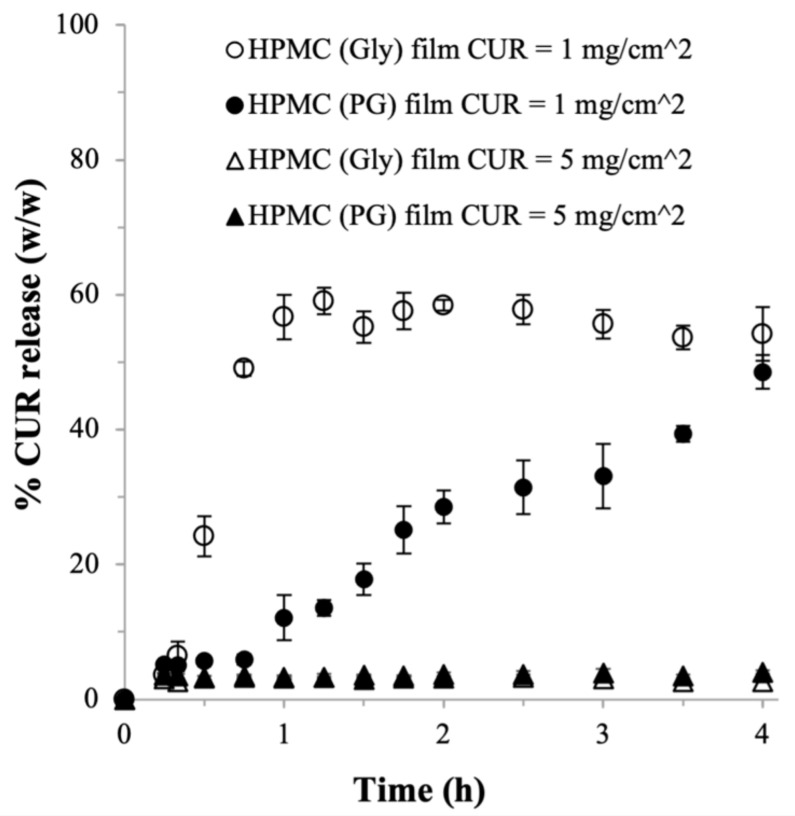
CUR release from HPMC (Gly) and HPMC (PG) films (*n* = 6, error bars represent the standard deviations).

For the HPMC (Gly) film prepared at theoretical CUR payload of 1 mg/cm^2^, the results of the dissolution tests in the SSF showed that roughly only 25% (*w*/*w*) of the CUR payload was released from the HPMC film (Gly) after 0.5 h. This signified the absence of a burst CUR release profile as expected from HPMC-based drug delivery systems (Figure 2). In this regard, the burst release is generally defined as rapid dissolution in which more than 85% (*w*/*w*) of the drug payload is released after 0.5 h [49]. The % CUR release then increased to reach approximately 60% after 1 h. However, the % CUR release plateaued afterwards to remain at around 60% after 4 h. As HPMC is a highly hydrophilic hydrogel, the incomplete CUR release was not likely to be caused by the lack of water uptake in certain segments of the HPMC (Gly) film, which would have suppressed the nanoplex dissolution in those segments. 

Therefore, we postulated that the incomplete CUR release was caused by precipitation of the supersaturated CUR concentration in the HPMC (Gly) film due to slow outward diffusion of the CUR molecules across the viscous gel layers. In a separate experiment, the HPMC (Gly) films were found to disintegrate very slowly in the SSF with disintegration time of 550 ± 17 min until its complete disintegration. The slow disintegration of the HPMC (Gly) film resulted in prolonged presence of intact gel layers that in turn slowed down the outward diffusion of CUR molecules. The slow diffusion rate caused the CUR supersaturation in the film to be at an unsustainably high level, resulting in high CUR precipitation propensity. Theoretically, the likelihood of the supersaturated CUR concentration in the film to precipitate increased with increasing CUR payload. This was evidenced experimentally by the severe inhibition of the CUR release from the HPMC (Gly) film prepared at theoretical CUR payload of 5 mg/cm^2^, where less than 5% of the CUR payload was released after 4 h (Figure 2). 

Compared to the HPMC (Gly) film, the HPMC (PG) film prepared at theoretical CUR payload of 1 mg/cm^2^ exhibited a significantly slower CUR release rate in the beginning with less than 15% (*w*/*w*) of the CUR payload was released after 1 h (Figure 2). Nevertheless, the CUR release picked up afterwards to reach around 50% (*w*/*w*) after 4 h. The slower CUR release in the HPMC (PG) film could be attributed to its thicker structure than the HPMC (Gly) film, resulting in longer diffusion pathways for the CUR molecules. Significantly, not unlike the finding in the HPMC (Gly) film, the HPMC (PG) film prepared at theoretical CUR payload of 5 mg/cm^2^ also exhibited severely inhibited CUR release (Figure 2). This was not unexpected, as the HPMC (PG) film also exhibited slow disintegration time at 520 ± 25 min. 

Importantly, the failures of both the HPMC (Gly) and HPMC (PG) films in releasing the CUR at high CUR payload led us to conclude that bare formulations of HPMC films were inadequate for high drug payload applications. Therefore, the effects of adding adjuvants to the HPMC films were investigated next. Gly was used as the plasticizer in the subsequent studies, as the results in Table 1 and Figure 2 showed the minimal role of the plasticizer’s type in the resultant characteristics of the HPMC films. Moreover, Gly was preferred over PG owing to its better cytotoxicity profile.

#### 2.2.2. Effects of Adjuvants

Besides precipitation of the supersaturated CUR concentration, another possible reason for the suppressed CUR release at high CUR payload was the aggregation of the nanoplex in the increasingly confined space of the film as the film solution was dried up. The aggregation limited the nanoplex’s surface areas exposed to the aqueous surrounding in the swollen polymer matrix, resulting in inhibited CUR release. Therefore, the effects of adding PVA—a widely used amphiphilic polymeric surfactant for colloids stabilization—were investigated to prevent the aggregation of the nanoplex in HPMC films. Moreover, PVA is also known for its good film-forming ability and mucoadhesive properties, rendering it a suitable adjuvant for buccal film applications [5]. 

In a separate study, the effects of adding AGN—another mucoadhesive polymer with good film-forming ability [50]—were investigated as the addition of anionic AGN to nonionic HPMC had been shown to improve the swelling properties of HPMC films [51]. We postulated that improved swelling properties of the HPMC film would lead to an improved CUR release profile. Physical characteristics of the HPMC–PVA and HPMC–AGN films prepared using Gly as the plasticizer are presented in Table 2. Both PVA and AGN were added at HPMC:PVA and HPMC:AGN mass ratios of 10:1. On this note, higher HPMC:PVA and HPMC:AGN ratios had also been investigated, but they were found to have significantly adverse effects on the film’s physical integrity and CUR entrapment efficiency; hence, they are not presented here. 

The results showed that the addition of PVA and AGN resulted in lower CUR entrapment efficiency from roughly 81–82% in their absence to 57–63% in their presence. This trend was observed at both theoretical CUR payloads of 1 and 5 mg/cm^2^. The lower CUR entrapment efficiency in the HPMC–PVA (Gly) and HPMC–AGN (Gly) films resulted in their lower experimental CUR payloads of around 0.6 and 3 mg/cm^2^ for theoretical CUR payloads of 1 and 5 mg/cm^2^, respectively. The impacts of adding PVA and AGN on the experimental CUR payloads of HPMC films were found to be similar. 

The lower CUR entrapment efficiency in the presence of PVA and AGN suggested that fewer CUR–CHI nanoplexes were embedded into the HPMC films in their presence. We postulated that the increased colloidal stability of the CUR–CHI nanoplex in the presence of PVA caused a larger proportion of the nanoplex to remain in the bulk fluid during the HPMC film formation, resulting in fewer nanoplexes incorporated into the film. For AGN, we postulated that AGN, being polyanions, interacted with the positively charged CUR–CHI nanoplex, resulting in destabilization of the nanoplex, where CUR was released from the nanoplex prematurely and in turn precipitated due to its low aqueous solubility. This resulted in less CUR available for incorporation into the HPMC film.

In terms of their weight and thickness, the HPMC–PVA (Gly) and HPMC–AGN (Gly) films were also comparable with each other, albeit the HPMC–AGN (Gly) film was slightly denser and thicker (Table 2). Both films also passed the weight and thickness variations at high CUR payload denoting the batch-to-batch consistency of their preparation. Compared to the bare HPMC (Gly) film, the weight and thickness of the HPMC–PVA (Gly) and HPMC–AGN (Gly) films were increased. 

**Figure 3:**
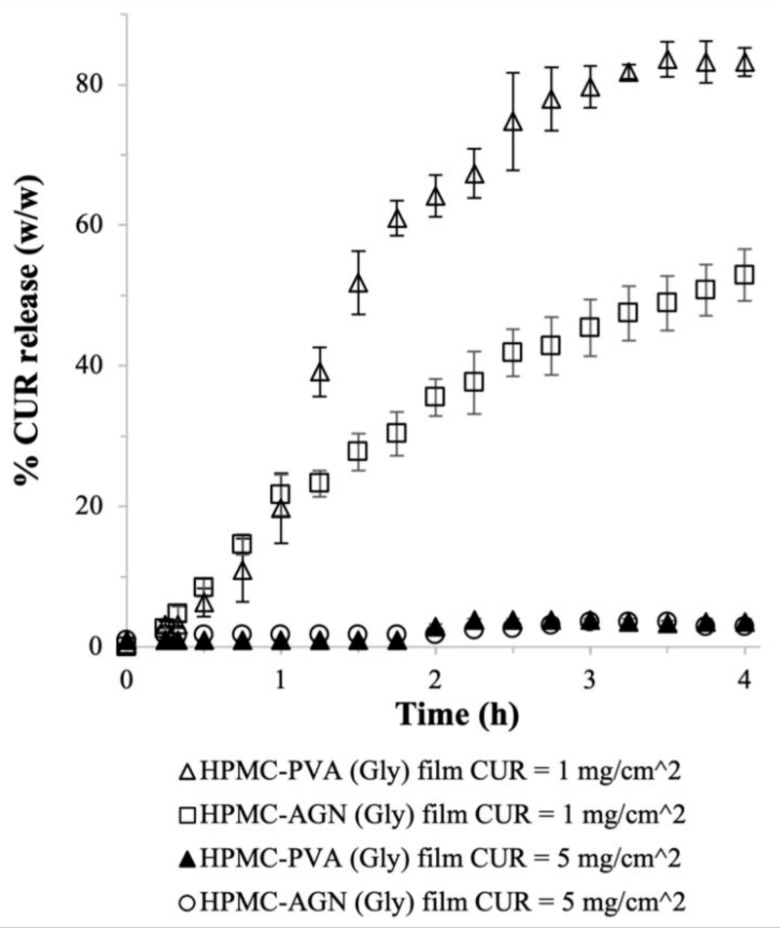
CUR release from HPMC–PVA (Gly) and HPMC–AGN (Gly) films (*n* = 6, error bars represent the standard deviations).

The lower CUR payloads obtained upon the addition of PVA and AGN would be acceptable if the CUR release from the films was enhanced compared to the CUR release from the bare HPMC film. Indeed, for the films prepared at theoretical CUR payload of 1 mg/cm^2^, the results showed that the CUR release from the HPMC–PVA (Gly) film was better than the CUR release from the bare HPMC (Gly) film (Figure 3). Specifically, the CUR release from the HPMC–PVA (Gly) film reached > 80% (*w*/*w*) after 4 h, in contrast to the plateau observed at ≈ 60% after 1 h for the HPMC (Gly) film (as shown earlier in Figure 2). The CUR release from the HPMC–PVA (Gly) film was slower in the beginning, where only roughly 20% (*w*/*w*) of the CUR payload was released after 1 h, resulting in a more sustained CUR release profile. Hence, the inclusion of long-chain PVA in the HPMC film was found to slow down the CUR release likely due to the increased physical barrier for outward molecular diffusion. 

A more sustained CUR release profile without a plateau after 1 h was also observed in the HPMC–AGN (Gly) film prepared at theoretical CUR payload of 1 mg/cm^2^ (Figure 3). In fact, the HPMC–AGN (Gly) film exhibited the ideal CUR release profile for sustained release with nearly zero-order kinetics. This could be attributed to the abovementioned improvement in the swelling properties of HPMC films with the addition of AGN. Importantly, the improvements in the CUR release profiles exhibited by the HPMC–PVA (Gly) and HPMC–AGN (Gly) films could compensate for their lower CUR entrapment efficiency. 

Unfortunately, despite the improved CUR release profiles at theoretical CUR payload of 1 mg/cm^2^, both HPMC–PVA (Gly) and HPMC–AGN (Gly) films remained unsuccessful in producing uninhibited CUR release at high theoretical CUR payload of 5 mg/cm^2^ (Figure 3). In this regard, even though our postulate that the presence of PVA could reduce the aggregation tendency of the nanoplex at high CUR payload might be true, the slower CUR release in the presence of PVA would keep the supersaturated CUR concentration in the HPMC film at an unsustainably high level, which increased the precipitation propensity of CUR in the film. The same phenomenon was believed to occur upon inclusion of AGN, which also slowed down the CUR release from the HPMC film. 

### 2.3. Starch-Based Films

#### 2.3.1. Physical Characteristics

The inability of the HPMC-based films to effectively release their CUR payloads upon an increase in the payload above 1 mg/cm^2^ required us to explore the use of an alternative mucoadhesive polymer, such as starch. Two types of starch were investigated, i.e., unmodified starch and HP starch. The latter is a modified starch in which some of the hydroxyl groups of the starch’s amylose and amylopectin molecules are substituted with hydroxypropyl groups at varying degree of substitution. HP starch is known to exhibit superior swelling and aqueous solubility than unmodified starch owing to its higher hydrophilicity [52]. The physical characteristics of the starch films prepared using Gly as the plasticizer are presented in Table 3. Like the HPMC films, both starch films were also prepared at two theoretical CUR payloads of 1 and 5 mg/cm^2^. 

The unmodified starch and HP starch films exhibited similar CUR entrapment efficiencies in the range of 70 to 80%. The CUR entrapment efficiency in the starch-based films was comparable, albeit slightly lower, to that of the bare HPMC films. Between the starch-based films prepared at theoretical CUR payloads of 1 and 5 mg/cm^2^, the difference in their CUR entrapment efficiencies were found to be statistically insignificant (Student’s *t*-test, *p* ≤ 0.05). Therefore, similar to the trend observed in the HPMC films, the effects of increasing the CUR payload had little effect on the CUR entrapment efficiency. This signified the ability of the starch-based films to accommodate the high CUR payload. 

With this CUR entrapment efficiency, the experimental CUR payloads of the starch-based films were determined to be approximately equal to 0.7 and 4 mg/cm^2^ at theoretical CUR payloads of 1 and 5 mg/cm^2^, respectively. The CUR payloads in the starch-based films were slightly lower than the CUR payloads in the bare HPMC films. The CHI contents in the starch-based films were approximately equal to 0.5 and 2.6 mg/cm^2^ at theoretical CUR payloads of 1 and 5 mg/cm^2^, respectively.

In terms of the weight and thickness, the unmodified starch films were denser and thicker than the HP starch films due to the higher density and viscosity of the unmodified starch. Specifically, the weight and thickness of the HP starch films were 44–50 mg/cm^2^ and 300–350 µm, respectively, compared to 65 mg/cm^2^ and 400–450 µm for the unmodified starch films. The thickness of the starch-based films was also shown to increase with increasing CUR payload indicating the accumulation of nanoplex as vertical layers in the starch films. Both starch films also passed the weight and thickness variation tests at high CUR payload, denoting their consistent preparation. 

In comparison to the HPMC films, the starch-based films were denser and thicker due to the higher concentration of the precursor solution required in the starch film to produce films with good physical integrity, i.e., 15% (*w*/*v*) for starch and 5% (*w*/*v*) for HPMC. On the one hand, the increased thickness exhibited by the starch-based films could affect patients’ experience upon administration of the buccal film either positively or negatively. This merits its own investigation in the future. On the other hand, the increased film thickness could bode well for the present goal of producing sustained CUR release profile at high CUR payload as we investigated in the next section. 

#### 2.3.2. CUR Release Profile

**Figure 4:**
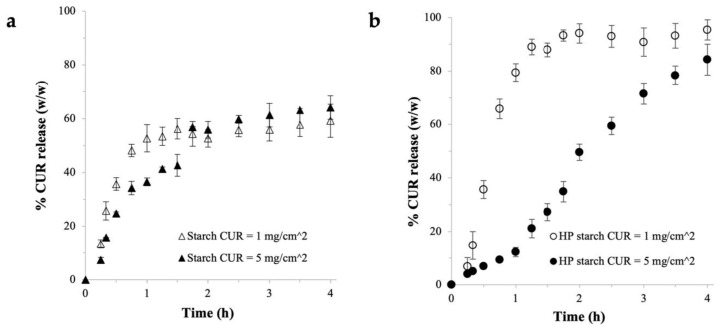
CUR release from (**a**) unmodified starch and (**b**) HP starch films (*n* = 6, error bars represent the standard deviations).

The CUR release profile from the unmodified starch film prepared at theoretical CUR payload of 1 mg/cm^2^ in Figure 4a was found to closely resemble that of the HPMC (Gly) film shown earlier in Figure 2. More specifically, the CUR release was relatively fast in the beginning, when approximately 52% (*w*/*w*) of the CUR payload was released from the unmodified starch film after 1 h. Afterwards, the CUR release slowed down greatly and reached a plateau at around 60% after 4 h. However, unlike the HPMC (Gly) film, the CUR release from the unmodified starch film was not severely suppressed at the high CUR payload. In fact, the percentage CUR release from the unmodified starch film prepared at theoretical CUR payload of 5 mg/cm^2^ was slightly higher after 4 h (Figure 4a). Nevertheless, the plateau in the CUR release at around 65% (*w*/*w*) remained evident after 2 h.

The CUR release was greatly improved in the HP starch films, as evidenced by the absence of a plateau in the CUR release profile (Figure 4b). For the HP starch film prepared at theoretical CUR payload of 1 mg/cm^2^, the initial CUR release was fast, with nearly 80% (*w*/*w*) of the CUR payload released after 1 h. The CUR release slowed down significantly afterwards to reach around 95% after 4 h. The CUR release from the HP starch film at this CUR payload thus did not exhibit the desired sustained CUR release profile. 

**Figure A1:**
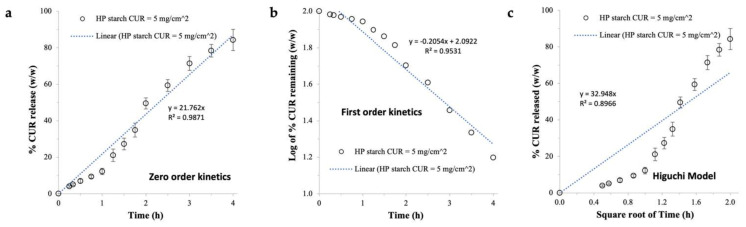
Dissolution kinetics modelling of the CUR release from HP starch film prepared at 5 mg/cm^2^ (**a**) zero order kinetics, (**b**) first order kinetics, and (**c**) Higuchi model.

In contrast, at theoretical CUR payload of 5 mg/cm^2^, the CUR release from the HP starch film was much slower, with roughly only 15% (*w*/*w*) of the CUR payload released after 1 h (Figure 4b). The CUR release rate picked up greatly afterwards with around 50% and 85% released after 2 h and 4 h, respectively. The HP starch film thus was able to produce the desired sustained release profile over 4 h at high CUR payload. The sustained CUR release profile fitted the zero-order kinetics closely, as shown in Figure A1 in Appendix A. Fitting the CUR release profiles to first-order kinetics and the Higuchi model led to poorer fitting, hence indicating that the CUR release from the starch-based film was independent of the CUR concentration in the film. Compared to the unmodified starch films, the superior CUR release observed in the HP starch films could be attributed to the aforementioned superior swelling and solubility of the HP starch, as well as the difference in the weight and thickness between the two films. 

Significantly, the results in Figure 4 established that starches were more suitable than HPMC for use as the buccal film matrix at high CUR payload. The ability of the starch-based films to produce uninhibited CUR release at high CUR payload suggested that the precipitation propensity of the supersaturated CUR concentration in the film was minimized. This occurred when the supersaturated CUR concentration generated by the nanoplex could diffuse out of the film in a timely manner. Compared to the HPMC films, both starch films, despite being thicker and denser, exhibited much shorter disintegration time of 360 ± 50 and 330 ± 25 min for the unmodified starch and HP starch, respectively. The shorter disintegration time enabled the entrapped CUR molecules to navigate the gel layers more quickly, resulting in faster outward diffusion of CUR. 

### 2.4. Further Characterizations of HP Starch Films

#### 2.4.1. FESEM and FTIR

**Figure 5:**
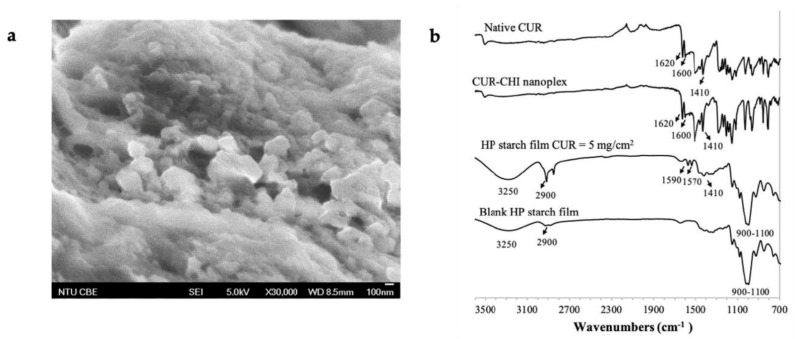
(**a**) FESEM; (**b**) FTIR spectra of the CUR–CHI nanoplex-loaded HP starch film.

**Figure A2:**
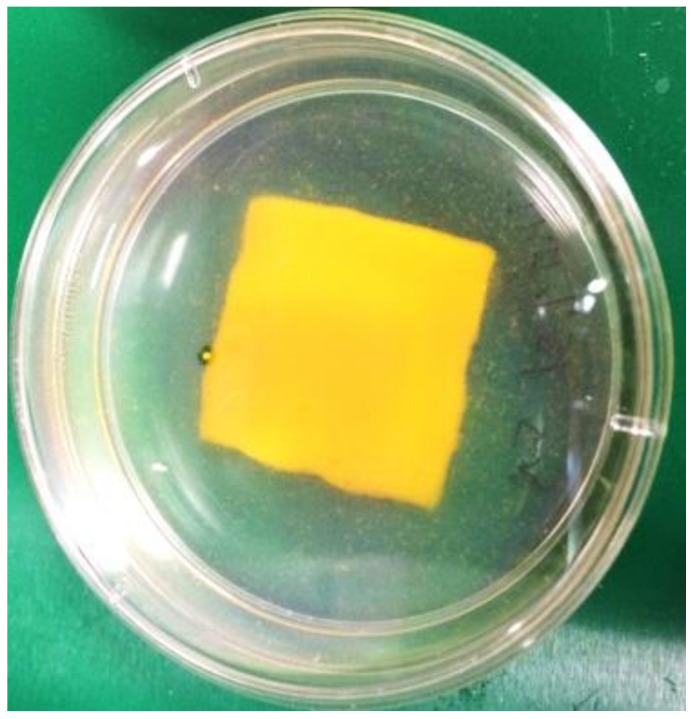
Macroscopic image of the CUR–CHI nanoplex-loaded HP starch film.

Having established the HP starch films as the optimal buccal film formulation at high CUR payload, further characterizations of the HP starch films were carried out. The macroscopic image of the HP starch film was presented in Figure A2 of Appendix A. The CUR–CHI nanoplex embedded in the HP starch films was visible in the FESEM image shown in Figure 5a using the HP starch film prepared at theoretical CUR payload of 5 mg/cm^2^ as the representative sample. The nanoplexes in the film were shown to be well dispersed as individual nanoparticles in the size range of 150–300 nm with minimal agglomeration among them. The FESEM image showed that the CUR–CHI nanoplex was well preserved upon its incorporation into the HP starch films. 

The presence of CUR in the HP starch film was verified by FTIR analysis via the appearance of the characteristic peaks of CUR at 1590, 1570, and 1410 cm^−1^ in the FTIR spectrum of the nanoplex-loaded HP starch film (Figure 5b). These three peaks were attributed to the (C=C) and (C=O) vibrations, C=C aromatic ring stretching vibration, and OH bending of the phenol group of CUR, respectively [19]. The three peaks in the HP starch films were shifted from higher wavenumbers of 1620, 1600, and 1410 cm^−1^ in the FTIR spectra of the native CUR and CUR–CHI nanoplex. These peaks were not visible in the FTIR spectrum of the blank HP starch film. The peaks at 900–1100, 2900, and 3250 cm^−1^ were attributed to the C-O-H bending, C-H stretching, and OH vibration of the amylose groups of the HP starch, respectively [53]. 

#### 2.4.2. CUR Payload Uniformity and Folding Endurance

The CUR payload uniformity among the independent samples of HP starch films (*n* = 10) prepared at theoretical CUR payloads of 1 and 5 mg/cm^2^ was examined (Table 4). The results showed that both HP starch films exhibited the CUR payload’s acceptance values (AV) equal to lower or slightly higher than the 15% maximum threshold value set by USP for AV. Hence, the HP starch films met the USP’s requirement for uniformity of a dosage unit [54]. Nevertheless, we recognized that the AV value at high CUR payload barely met the acceptance limit; thus, improvements in the HP starch film formulation will be needed in the future. In terms of their physical robustness, both HP starch films exhibited good folding endurance with values around of 2.5 to 2.7 denoting no film breakage was observed after ≥300 double folds (Table 4).

#### 2.4.3. Amorphous form Stability and Thermal Stability

**Figure 6:**
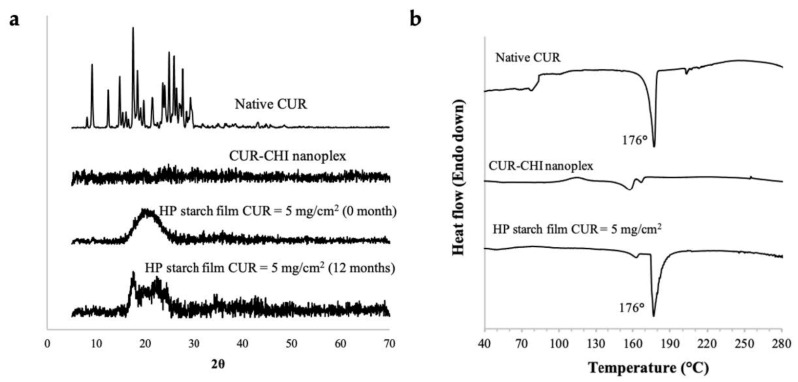
(**a**) PXRD; (**b**) DSC of the CUR–CHI nanoplex-loaded HP starch film.

PXRD analysis of the nanoplex-loaded HP starch film performed after the accelerated storage did not show the appearance of strong intensity peaks, which were present in the PXRD pattern of the native CUR crystals (Figure 6a). The HP starch film prepared at theoretical CUR payload of 5 mg/cm^2^ was used as the representative sample for PXRD. Thus, the CUR–CHI nanoplex in the HP starch film maintained its amorphous form after the accelerated storage equivalent to twelve-month storage at ambient condition. Nevertheless, the amorphous halo at 2θ≈ 15–25° visible in the PXRD pattern of the HP starch film before storage became less pronounced after storage. The amorphous halo was replaced by low-intensity peaks, indicating decreased amorphous contents as crystallization of some of the nanoplex took place during storage. 

**Figure A3:**
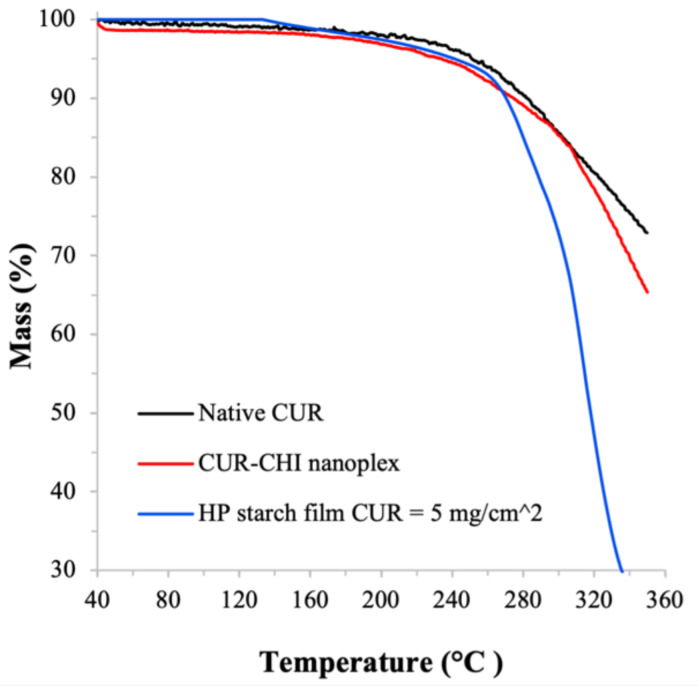
TGA of the HP starch film prepared at theoretical CUR payload of 5 mg/cm^2^.

The TGA results showed that the native CUR and HP starch film started to decompose at temperatures above 280 °C (Figure A3 in Appendix A); thus, the DSC thermograph for thermal stability was analyzed at temperatures below the decomposition temperature. DSC thermograph of the HP starch film prepared at theoretical CUR payload of 5 mg/cm^2^ showed the appearance of a sharp endothermic peak at around 176 °C, which was typical of the melting point of crystalline CUR (Figure 6b). Not unexpectedly, the same peak at 176 °C appeared in the DSC thermograph of the native CUR crystals. The DSC results indicated that the CUR–CHI nanoplex in the HP starch film experienced amorphous to crystalline transition upon heating above 170 °C. In contrast, the melting point peak was not evident in the DSC thermograph of the free CUR–CHI nanoplex, where only solid transition events at around 160–170 °C were recorded. This signified the higher thermal stability of the free CUR–CHI nanoplex compared to the nanoplex embedded in the film. Nevertheless, the CUR–CHI nanoplex embedded in the HP starch film remained stable upon heating up to 150 °C, hence, the drying step in the film preparation should not adversely affect the amorphous form stability of the CUR–CHI nanoplex. 

## 3. Materials and Methods

### 3.1. Materials

*Materials for CUR–CHI nanoplex’s preparation and characterization*: curcumin (CUR) from turmeric rhizome (>95% curcuminoid content) was purchased from Alfa Aesar (Singapore). Chitosan (CHI) (190–310 kDa, 75–85% deacetylation), potassium hydroxide (KOH), disodium phosphate (Na_2_HPO_4_.7H_2_O), ethanol, potassium dihydrogen phosphate (KH_2_PO_4_), sodium chloride (NaCl), hydrogen chloride (HCl), phosphoric acid (H_3_PO_4_), and glacial acetic acid (AA) were purchased from Sigma Aldrich (Singapore). *Materials for buccal film’s preparation*: hydroxypropyl methylcellulose (HPMC) (MW = 26 kDa), polyvinyl alcohol (PVA) (90 kDa, 99% hydrolyzed), sodium alginate (AGN), glycerol (Gly), and propylene glycol (PG) were purchased from Sigma Aldrich (Singapore). Hydroxypropyl (HP) starch (LYCOAT^®^ NG720) and pre-gelatinized starch (LYCATAB^®^) were generously provided by Roquette (Singapore). 

### 3.2. Methods

#### 3.2.1. Preparation and Characterization of CUR–CHI Nanoplex

The amorphous CUR–CHI nanoplex was prepared following the protocols presented in Lim et al. [19]. Briefly, CUR was dissolved at 5 mg/mL in 0.1M KOH (pH 13) and separately, CHI was dissolved at 5.9 mg/mL in 1.2% (*w*/*v*) AA (pH 2.7), both at room temperature. Equal volumes of the CUR and CHI solutions (10 mL each) were then mixed immediately after their preparation under gentle stirring to minimize alkaline degradation of CUR. The resultant CUR–CHI suspension was ultrasonicated for 15 s at 20 kHz (VC 505, Sonics, New Town, CT, USA) to break up large agglomerates (if any). The nanoplex suspension then underwent two cycles of ultracentrifugation (14,000× *g*, 10 min) and washing with deionized water to remove free CUR and free CHI that did not form the nanoplex. The CUR encapsulation efficiency into the nanoplex was characterized by measuring the free CUR concentration in the supernatant after the first centrifugation step using high performance liquid chromatography (HPLC) as described below. Afterwards, the washed CUR–CHI nanoplex suspension was lyophilized for 24 h at −52 °C and 0.05 mbar in Alpha 1–2 LD Plus freeze dryer (Martin Christ, Osterode am Harz, Germany) for characterization purposes.

**Figure A4:**
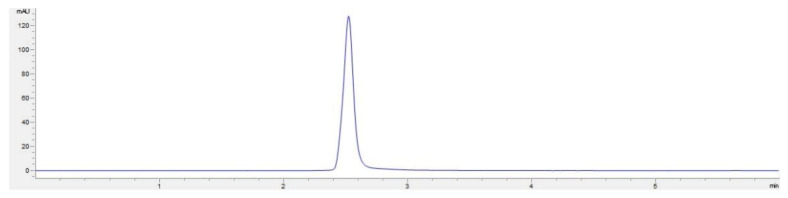
HPLC chromatogram of CUR detection.

The size and zeta potential of the CUR–CHI nanoplex suspension were characterized in triplicates by dynamic light scattering (DLS) after 100× dilution, using Brookhaven 90 Plus Nanoparticle Size Analyzer (Brookhaven Instruments Corporation, Holtsville, NY, USA). The CUR content in the nanoplex, which was defined as the amount of CUR per unit mass of the CUR–CHI nanoplex, was determined in triplicates by dissolving 1 mg of the lyophilized nanoplex powder in 10 mL 80% (*v*/*v*) ethanol. The amount of CUR in the ethanol was subsequently determined by HPLC (Agilent 1100, Agilent Technologies, Santa Clara, CA, USA) at CUR detection wavelength of 423 nm. The HPLC was performed using ZORBAX Eclipse Plus C18 column (250 × 4.6 mm, 5 µm particle size) and 75% (*v*/*v*) acetonitrile solution as the mobile phase at flow rate of 1 mL/min, resulting in CUR retention time of approximately 2.8 min. The HPLC chromatogram of the CUR detection was presented in Figure A4 of Appendix A. The physical appearances of the CUR–CHI nanoplex before and after incorporation into the buccal film were examined by field emission scanning electron microscope (FESEM) (JSM 6700F, JEOL, Peabody, MA, USA). 

#### 3.2.2. Preparation of CUR–CHI Nanoplex-Loaded Buccal Film

The precursor solution for the HPMC film was prepared by overnight dissolution of HPMC in deionized water at 5% (*w*/*v*) under gentle stirring. The plasticizer, Gly or PG, was added to the HPMC solution at 5% (*w*/*w*). In the study on the effects of adjuvants inclusion, aqueous AGN or PVA solution was added to the HPMC + plasticizer solution at 0.5% (*w*/*v*). Freshly prepared CUR–CHI nanoplex was added last at theoretical CUR payloads of 1 or 5 mg CUR per cm^2^ of the film. The precursor solution was then vortexed for 1 min to ensure its homogeneity. Afterwards, the precursor solution was casted onto a 9 cm diameter glass petri dish at a liquid height of 4 mm. Next, the petri dish was transferred to a convective laminar flow oven for drying at 60 °C for 3 h. The resultant dried HPMC film was peeled off the petri dish and stored in a sealed plastic bag for characterizations. The starch films were prepared by the same procedures at starch concentrations of 15% (*w*/*v*) for both unmodified and HP starches using 5% (*w*/*w*) Gly as the plasticizer for both. The HP starch was pre-gelatinized in deionized water at 80 °C prior to the film preparation. The CUR–CHI nanoplex was also added at theoretical CUR payloads of 1 and 5 mg CUR per cm^2^ of the starch films. 

#### 3.2.3. Experimental CUR Payload, CUR Payload Uniformity, CUR Entrapment Efficiency

The experimental CUR payload in the buccal film (in mg of CUR per cm^2^ of film) was determined by dissolving a 2 × 2 cm^2^ square film samples in 25 mL 80% (*w*/*v*) ethanol solution for 1 h. Afterwards, the amount of CUR dissolved in the ethanol was determined by HPLC as previously described. The CUR payloads of ten independently prepared films were determined from which the CUR payload uniformity was characterized. According to the United States Pharmacopeia’s (USP) criteria, the payload uniformity of a drug dosage was deemed acceptable when the acceptance value (AV) was less than 15%. The definition for AV is elaborately explained in USP monograph “<905> Uniformity of Dosage Units” [54] and not repeated here for brevity. After the experimental CUR payload was determined, the CUR entrapment efficiency (*n* = 10) was calculated from the ratio of the experimental CUR payload to the theoretical CUR payload.

#### 3.2.4. Weight, Thickness, Folding Endurance of the Buccal Film

The weight and thickness of the buccal film were characterized from 9 cm diameter circular films using analytical balance and digital caliper, respectively. The variations in the buccal film’s weight and thickness were characterized using ten independently prepared films. According to the USP’s criteria, the variations in the weight and thickness among the independent samples were determined to be acceptable if each sample exhibited weight (or thickness) within ±10% of the arithmetic average of the weight (or thickness) [54]. The folding endurance was defined as the logarithmic (log10) of the number of double folds that resulted in the breakage of the buccal film. Briefly, triplicates of 2 × 2 cm^2^ square film samples were manually double folded and the number of folds at which the film started showing signs of breakage was noted. 

#### 3.2.5. CUR Dissolution from Buccal Film and Film Disintegration

CUR dissolution from the buccal film was characterized in six replicates in simulated saliva fluid (SSF) under a sink condition at ¼ of the thermodynamic saturation solubility of CUR in the SSF (C_Sat_). The SSF was prepared following the formulation of Peh and Wong [43] in which 4.49 g of Na_2_HPO_4_.7H_2_O, 0.19 g KH_2_PO_4_, and 8.00 g NaCl were dissolved in one 1 L of deionized water. The pH of the SSF was adjusted to pH 6.75 by the addition of H_3_PO_4._ C_Sat_ of CUR in the SSF was experimentally determined by incubating excess CUR in 100 mL SSF maintained at 37 °C in a shaking incubator. After 24-h incubation, the CUR concentration in the SSF was determined by HPLC as previously described, resulting in C_Sat_ equal to 6.2 µg/mL. 

Briefly, 2 × 2 cm^2^ square film samples were completely immersed in 100 mL SSF maintained at 37 °C in a shaking incubator. At specific timepoints over a 4-h period, 1 mL of aliquot was withdrawn from the dissolution flask and the same volume of fresh SSF was added back to the flask as replenishment. The aliquot was syringe filtered (0.22 pore size), followed by 3-min ultracentrifugation at 14,000× *g*. Afterwards, the CUR concentration in the supernatant was determined by HPLC as previously described. 

In a separate experiment, the disintegration time of the buccal film was characterized in triplicates by immersing 2 × 2 cm^2^ square film samples in 10 mL SSF in a petri dish maintained at 37 °C in a shaking incubator. The smaller volume of SSF used in the disintegration test was to simulate the aqueous environment of oral cavity more closely. Herein, the disintegration time was defined as the time at which the buccal film had been completely disintegrated to aqueous suspension of the nanoplex and polymers, where no visible film fragments were present. 

#### 3.2.6. PXRD, DSC, and FTIR

The long-term stability of the amorphous form of the CUR–CHI nanoplex in the buccal film was examined by storing the nanoplex-loaded film in an open container placed inside a desiccator for three months under accelerated storage condition of 40 °C and 75% relative humidity. The accelerated storage condition was approximately equivalent to twelve-month storage under ambient condition (i.e., 25 °C and 60% relative humidity). The 75% relative humidity was generated inside the desiccator by placing an open container of saturated NaCl solution at 40 °C. At the end of the storage, the amorphous form of the nanoplex in the buccal film was examined by powder X-ray diffraction (PXRD) using D8 Advance X-ray Diffractometer (Bruker, Berlin, Germany) performed between 10° and 70° (2θ) with a step size of 0.02°/s. For comparison, the PXRD analysis was also carried out for the native CUR, the free CUR–CHI nanoplex, and the nanoplex-loaded film before storage. 

The thermal stability of the amorphous form of the CUR–CHI nanoplex in the buccal film was characterized by thermal gravimetric analysis (TGA) (Pyris Diamond TGA, PerkinElmer, Waltham, MA, USA) and differential scanning calorimetry (DSC) (DSC 822E, Mettler Toledo, Columbus, OH, USA). The TGA analysis was performed at heating rate of 10 °C/min between 30 °C and 400 °C. The DSC analysis was performed at heating rate of 2 °C/min between 30 °C to 300 °C. Lastly, the presence of CUR in the buccal film was verified by Fourier transform infrared spectroscopy (FTIR) from 450 and 4000 cm^−1^ at 4 cm^−1^ spectral resolution in Spectrum One (Perkin–Elmer, Waltham, MA, USA). The DSC/TGA and FTIR analyses were also performed for the native CUR and the free CUR–CHI nanoplex. 

#### 3.2.7. Statistical Analysis

All experiments were performed with a minimum of three replicates and the results are presented as mean ± standard deviation. The statistical significance was analyzed using Student’s *t*-test in GraphPad Prism software (GraphPad Software, San Diego, CA, USA). All *p*-values were two-sided and considered significant at *p* ≤ 0.05, unless stated otherwise.

## 4. Conclusions

The feasibilities of HPMC and pre-gelatinized starch-based films as buccal sustained release delivery systems of amorphous CUR–CHI nanoplex at high CUR payload were investigated. HPMC and starch films were found to exhibit similar CUR entrapment efficiencies (≈ 80% *w*/*w*), resulting in their similar CUR payloads in the range of 0.6 to 4 mg/cm^2^. Both HPMC and starch films were able to accommodate higher CUR payloads without any adverse effect on the CUR entrapment efficiency. The starch films were denser and thicker than HPMC films, as higher film precursor concentrations were needed to produce starch films with good physical integrity. Despite being denser and thicker, starch films disintegrated faster than HPMC films. The faster disintegration time of the starch films resulted in its significantly superior CUR release profiles at high CUR payload (4 mg/cm^2^) compared to the HPMC films. This was because the faster disintegration time enabled faster outward CUR molecular diffusion across the swollen polymer matrix, which in turn reduced the precipitation propensity of the highly supersaturated CUR concentration generated in the film by the amorphous nanoplex dissolution. The superior CUR release exhibited by the starch films, nevertheless, were not as evident at lower CUR payloads (≤1 mg/cm^2^) due to the lower CUR supersaturation level generated in the film. Between the unmodified starch and HP starch films, the HP starch films exhibited superior CUR release profiles in which at least 85% (*w*/*w*) of the CUR payload was released within 4 h, whereas the CUR release from the unmodified starch plateaued at around 65% (*w*/*w*) after the same period. At the high CUR payload, HP starch films exhibited the ideal sustained CUR release profile following the zero-order kinetics. The films prepared from different batches (*n* = 10) exhibited good CUR payload uniformity and minimal weight/thickness variations. The HP starch films were physically robust with high folding endurance and the embedded nanoplex was thermally stable with good long-term storage stability. 

## Figures and Tables

**Table 1:** Physical characteristics of HPMC (Gly) and HPMC (PG) films (*n* = 10, mean ± stdev).

Type of Film	Theoretical CUR Payload (mg/cm^2^)	% CUR Entrapment (*w*/*w*)	CUR Payload (mg/cm^2^)	Weight (mg/cm^2^)	Thickness (µm)
HPMC (Gly)	1	81 ± 3	0.8 ± 0.03	13 ± 1	110 ± 3
HPMC (Gly)	5	82 ± 5	4 ± 0.3	30 ± 1	231 ± 7
HPMC (PG)	1	83 ± 3	0.8 ± 0.03	17 ± 2	138 ± 6
HPMC (PG)	5	79 ± 8	4 ± 0.4	31 ± 1	254 ± 5

**Table 2:** Physical characteristics of the HPMC–PVA (Gly) and HPMC–AGN (Gly) films (*n* = 10, mean ± stdev).

Type of Film	Theoretical CUR Payload (mg/cm^2^)	% CUR Entrapment (*w*/*w*)	CUR Payload (mg/cm^2^)	Weight (mg/cm^2^)	Thickness (µm)
HPMC–PVA (Gly)	1	59 ± 1	0.6 ± 0.01	16 ± 1	130 ± 5
HPMC–PVA (Gly)	5	57 ± 9	3 ± 0.4	24 ± 1	194 ± 5
HPMC–AGN (Gly)	1	58 ± 1	0.6 ± 0.01	20 ± 2	159 ± 15
HPMC–AGN (Gly)	5	63 ± 2	3 ± 0.1	27 ± 3	230 ± 5

**Table 3:** Physical characteristics of the unmodified starch and HP starch films (*n* = 10, mean ± stdev).

Type of Film	Theoretical CUR Payload (mg/cm^2^)	% CUR Entrapment (*w*/*w*)	CUR Payload (mg/cm^2^)	Weight (mg/cm^2^)	Thickness (µm)
Starch	1	74 ± 5	0.7 ± 0.05	65 ± 2	404 ± 7
Starch	5	79 ± 5	4 ± 0.2	65 ± 4	453 ± 23
HP starch	1	74 ± 3	0.7 ± 0.03	44 ± 1	307 ± 5
HP starch	5	77 ± 6	4 ± 0.3	51 ± 2	352 ± 10

**Table 4:** CUR payload uniformity (*n* = 10) and folding endurance of HP starch films (*n* = 3).

CUR Payload (mg/cm^2^)	AV for CUR Payload (%)	Folding Endurance
1	13.0	2.5 ± 0.07
5	15.2	2.7 ± 0.04

## Data Availability

Not applicable.

## Abbreviations

AA	Acetic acid
AGN	Alginate
C_Sat_	Thermodynamic saturation solubility
CHI	Chitosan
CUR	Curcumin
DLS	Dynamic light scattering
DSC	Differential scanning calorimetry
FESEM	Field emission scanning electron microscope
FTIR	Fourier transform infrared spectroscopy
Gly	Glycerol
HPMC	Hydroxypropyl methyl cellulose
HP	Hydroxypropyl
PG	Propylene glycol
PXRD	Powder X-ray diffraction
PVA	Polyvinyl alcohol
SSF	Simulated saliva fluid
TGA	Thermal gravimetry analysis
USP	United States Pharmacopeia
